# Two Novel Mutations in Myosin Binding Protein C Slow Causing Distal Arthrogryposis Type 2 in Two Large Han Chinese Families May Suggest Important Functional Role of Immunoglobulin Domain C2

**DOI:** 10.1371/journal.pone.0117158

**Published:** 2015-02-13

**Authors:** Xuefu Li, Bomeng Zhong, Weitian Han, Ning Zhao, Wei Liu, Yu Sui, Yawen Wang, Yongping Lu, Hong Wang, Jianxin Li, Miao Jiang

**Affiliations:** 1 Key Laboratory of Reproductive Health of Liaoning Province, Shenyang, China; 2 Emergency department, Nanjing First Hospital, Nanjing, China; NIDCR/NIH, UNITED STATES

## Abstract

Distal arthrogryposes (DAs) are a group of disorders that mainly involve the distal parts of the limbs and at least ten different DAs have been described to date. DAs are mostly described as autosomal dominant disorders with variable expressivity and incomplete penetrance, but recently autosomal recessive pattern was reported in distal arthrogryposis type 5D. Mutations in the contractile genes are found in about 50% of all DA patients. Of these genes, mutations in the gene encoding myosin binding protein C slow *MYBPC1* were recently identified in two families with distal arthrogryposis type 1B. Here, we described two large Chinese families with autosomal dominant distal arthrogryposis type 2(DA2) with incomplete penetrance and variable expressivity. Some unique overextension contractures of the lower limbs and some distinctive facial features were present in our DA2 pedigrees. We performed follow-up DNA sequencing after linkage mapping and first identified two novel *MYBPC1* mutations (c.1075G>A [p.E359K] and c.956C>T [p.P319L]) responsible for these Chinese DA2 families of which one introduced by germline mosacism. Each mutation was found to cosegregate with the DA2 phenotype in each family but not in population controls. Both substitutions occur within C2 immunoglobulin domain, which together with C1 and the M motif constitute the binding site for the S2 subfragment of myosin. Our results expand the phenotypic spectrum of MYBPC1-related arthrogryposis multiplex congenita (AMC). We also proposed the possible molecular mechanisms that may underlie the pathogenesis of DA2 myopathy associated with these two substitutions in MYBPC1.

## Introduction

Distal arthrogryposis(DA) is a group of disorders that mainly involve the distal parts of the limbs and are characterized by congenital contractures of two or more different body areas [1]. Since the Hall’s classification of DA was revised [1,2], at least ten different forms of DA (DA1-DA10) have been reported and distal arthrogryposes (DAs) were mostly described as autosomal dominant disorders, but recently autosomal recessive pattern was reported in distal arthrogryposis type 5D(DA5D) [3]. In the gene discovery studies, DA1 (MIM 108120), DA2B (Sheldon-Hall syndrome [SHS], MIM 601680) and DA2A (Freeman-Sheldon syndrome [FSS], MIM 193700) were suggested most common DAs. DA1, DA2B/SHS and DA2A/FSS share some major diagnostic criteria. However, they can be distinguished from one another based on diagnostic criteria, which include the absence of facial contractures in most individuals with DA1, the presence of mild to moderate facial contractures in SHS [4] and the presence of moderate to severe facial contractures in FSS. Nevertheless, making the distinction between SHS and FSS based on clinical characteristics alone is so challenging that Stevenson and his colleagues proposed a strict diagnostic criteria for FSS. In contrast to individuals with classical FSS, patients with SHS have a larger oral opening, a triangular face with small pointed chin and lack an H-shaped dimpling of the chin (H-chin) [5,6]. Additional features commonly found in FSS include scoliosis, prominent superciliary ridge, blepharophimosis, potosis, strabismus, dental crowding, hypoplastic alae nasi, a long philtrum, and feeding difficulty at birth [2,5,7].

In the last two decades, the majority of the genes implicated in autosomal dominant DA encode components of the sarcomere or contractile apparatus of myofibers, including β-tropomyosin (TPM2), troponin I type 2 (TNNI2), troponin T type 3 (TNNT3), myosin heavy chain 3 (MYH3)[6,8–11] and myosin-binding protein C1 (MYBPC1) [12]. Recently, mutations in endothelin-converting enzyme-like 1 (ECEL1) gene, which encodes a neuronal endopeptidase and is expressed in the brain and peripheral nerves, were found to be responsible for nearly 88%(15/17) of the reported autosomal recessive DA5D families[3,13–15]. Mutations in piezo-type mechanosensitive ion channel component 2 (PIEZO2), which together with PIEZO1 are recently identified, widely expressed, mechanically activated ion channels that are hypothesized to play a role in mechanotransduction in mammals, could explain about 84% (26/31) of the reported autosomal dominant DA5D families and 83%(10/12) of the reported DA3 families[16–18]. Mutations in the contractile genes were found in about 50% of all DA patients [13] and mostly in DA1, DA2B and DA2A. Of these genes, mutations in *MYH3* are the most common known cause of distal arthrogryposis [6]. However, only two *MYBPC1* missense mutations were reported in two DA1B families [12].

**Fig 1:**
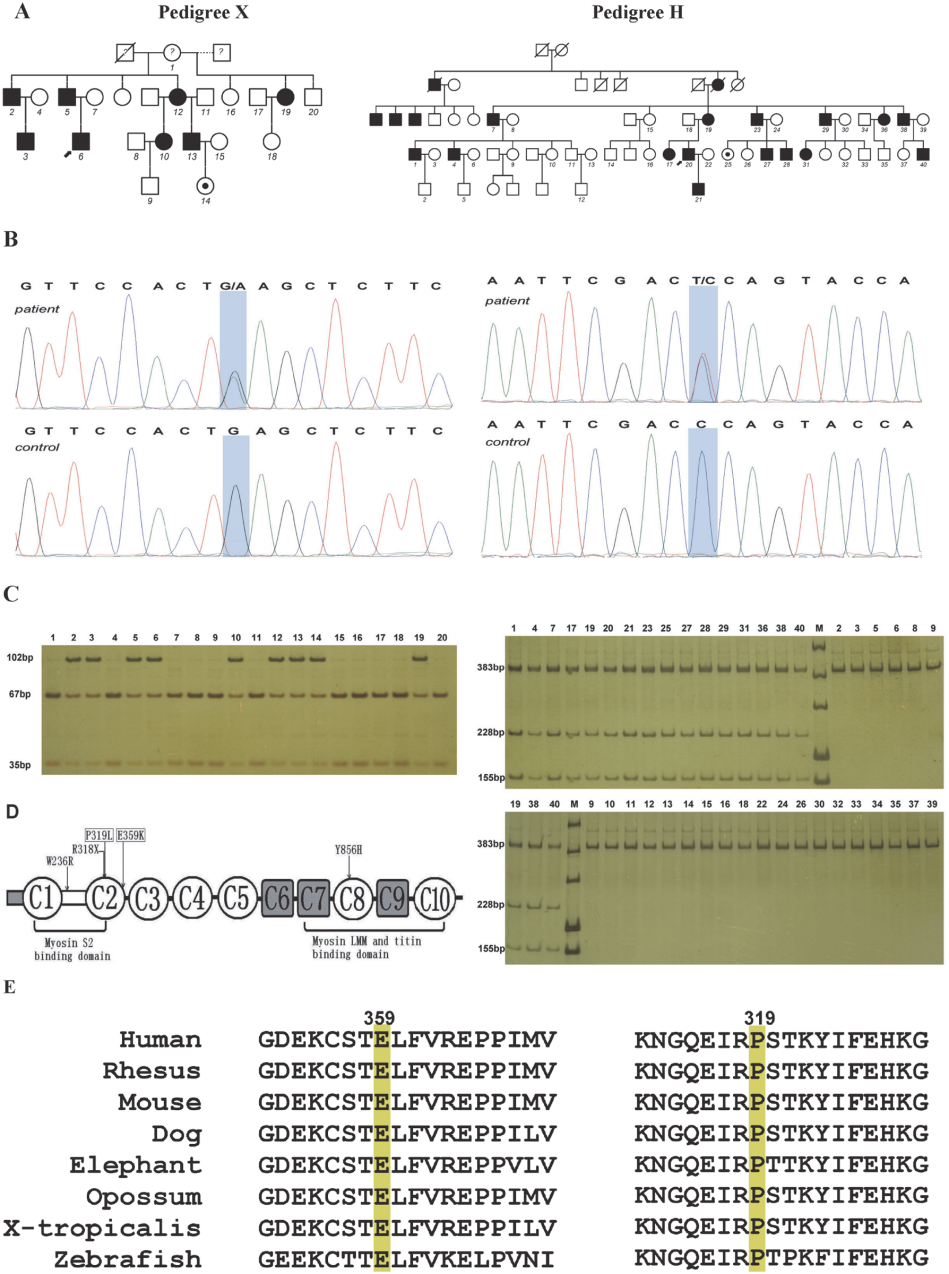
*MYBPC1* mutations in the DA2 families. **A**: Chinese kindreds affected with DA2. Solid and open symbols represent affected and unaffected individuals, respectively. The small filled circle inside the open circle represents an asymptomatic carrier. The question mark indicates the unknown status of the individuals of family X. The numbers denote individuals whose DNA samples were available for the analysis. Dotted line in pedigree X indicates individuals 16, 19 and 20 were born from different fathers. **B**: The c. 1075G>A (p.E359K) mutation in pedigree X and c. 956C>T (p.P319L) mutation in pedigree H of *MYBPC1*. Sanger sequence analysis of an affected individual and a normal unaffected control. The mutations shown as a light blue shadow each. **C**: Digests of *MYBPC1* amplicons from family members. In pedigree X, the mutation eliminates an Ecl136II restriction site. Digests of *MYBPC1* amplicons (102 bp) from affected individuals fractionate into three fragments (102 bp, 67 bp and 35 bp), whereas only two fragments (67 bp and 35 bp) were found in the unaffected members and the mildly symptomatic founder (X1). In pedigree H, the mutation creates a novel Hinf I restriction site. Digests of *MYBPC1* amplicons (421 bp) from affected individuals fractionate into four fragments (383 bp, 228 bp, 155 bp, and 38 bp), whereas the 38-bp product cannot be observed on this gel. Only one fragment (383 bp) is observed in unaffected individuals. M indicates DNA marker. **D**: Schematic demonstrating the position of the mutations in the MYBPC1 protein. Two DA2 *MYBPC1* mutations reported in the present study, p.P319L and p.E359K are shown in rectangles. Similar to the mutation of R318X found in patients with lethal congenital contracture syndrome type 4, both of them are located within the C2 immunoglobulin domain. The immunoglobulin domains C1, C2 and the MyBP-C unique motif (M-motif) between them constitute a critical region, which has been shown to interact with myosin S2. The DA1 *MYBPC1* mutations p.W236R and p.Y856H, located within the M-motif and the C8 repeat respectively, are also shown. Immunoglobulin domains are shown as open circles and fibronectin type III domains as grey squares. M-motif between C1 and C2 domain is shown as open rectangle. **E**: Amino acid alignment around the each affected residue of the MYBPC1 protein. The highly conserved E359 and P319 are highlighted in yellow.

Myosin binding protein C (MyBP-C) consists of a family of thick filament associated proteins and it contributes to the regular organization and stabilization of thick filaments and modulates the formation of cross-bridges between myosin and actin [19]. The core structure of MyBP-C is composed of seven immunoglobulin (Ig) domains and three fibronectin type III (Fn-III) repeats, numbered from the NH2-terminus as C1–C10. The C1 domain is flanked by two unique motifs, one enriched in proline and alanine residues, termed Pro/Ala rich motif and a conserved linker, referred to as M motif (Fig. 1)[20]. Three isoforms of MyBP-C exist in striated muscles: cardiac, slow skeletal, and fast skeletal. To date, much of our knowledge on MyBP-C originates from most studies that have focused on the cardiac form, due to its direct involvement in the development of hypertrophic cardiomyopathy. However, research into MYBPC1 is limited, due to only three *MYBPC1* mutations reported in human disease [12,21].

In this study, we performed follow-up DNA sequencing after linkage mapping in two large Chinese DA2 families and report two novel missense mutations in *MYBPC1* to be involved in DA2. One of the DA kindreds was introduced by germline mosaicism of the de novo *MYBPC1* mutation. Our results suggest the immunoglobulin domain C2 of MYBPC1 may play an important role in binding to S2 fragment of myosin. We also suggest the possible molecular mechanisms that may underlie the pathogenesis of DA2 myopathy associated with these two substitutions in MYBPC1.

## Materials and Methods

### Patients

**Fig 2:**
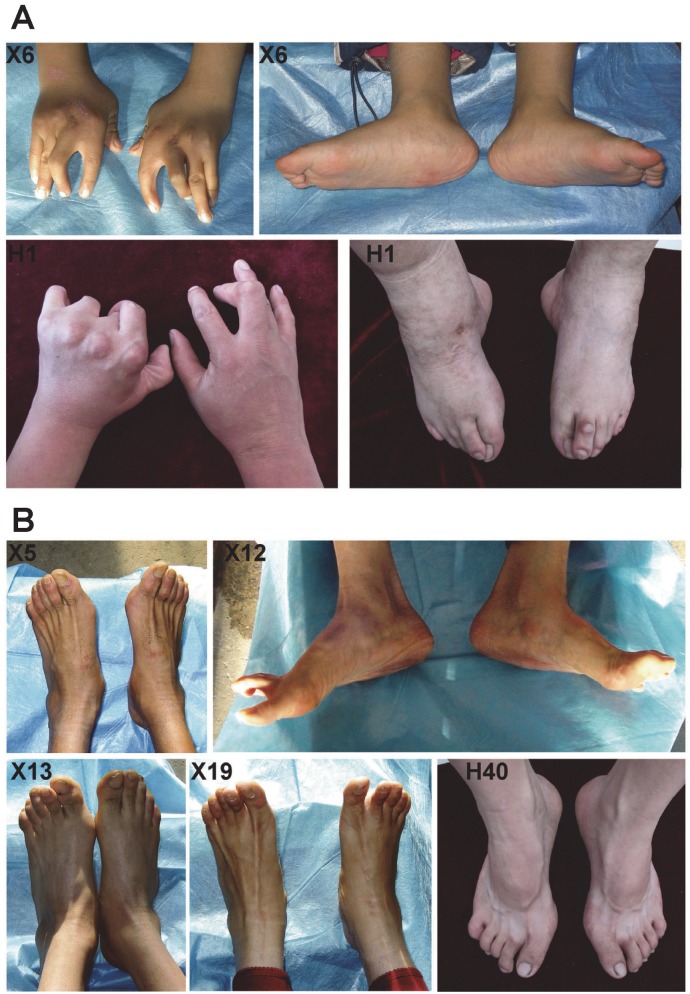
Typical limb contractures and overextension contractures in the two DA2 families. **A**: Note that typical limb contractures include ulnar deviation of fingers, adducted stiff/clasped thumbs, severe camptodactyly, overlapping fingers (H1), vertical talus(X6) and clubfeet after surgical corrections accompanied by overriding toes (H1) in severely affected individuals. **B**: Note that overextension contractures were observed at the metatarsophalangeal joints(X5, X12 and H40) or the proximal interphalangeal joints(X13 and X19) of the toes.

These two autosomal dominant Han Chinese DA families (pedigrees X and H) are from Northeast China and each kindred has multiple male and female affected individuals across four or five generations with incomplete penetrance and variable expressivity (Fig. 1). Individuals were considered as affected if they had at least one major diagnostic criterion in the context of an affected family. Eight affected individuals and one asymptomatic carrier in family X were available for clinical evaluation, while seven affected individuals and one asymptomatic carrier in family H were included in the investigation. Clinical findings in these two families include ulnar deviation of fingers, adducted stiff/clasped thumb, camptodactyly and hypoplastic and/or absent flexion creases in the upper limbs and overriding toes, flexed toes and planovalgus in the lower limbs (details in Fig. 2 and Table 1). These clinical findings meet the major diagnostic criteria for DA. Notably, overextension contractures were observed at the metatarsophalangeal joints or the proximal interphalangeal joints of the toes of 4 affected individuals in family X and one in family H (Fig. 2 and Table 1).

**Table 1:** Clinical data of affected individuals in these two Chinese families with DA2.

Clinical findings			Kindred X									Kindred H						
			X1	X2	X3	X5	X6	X10	X12	X13	X19	H1	H19	H23	H29	H36	H38	H40
**Facial features**		Face shape	**O**	**R**	**T**	**T**	**S**	**O**	**O**	**O**	**S**	**S**	**S**	**R**	**S**	**D**	**R**	**O**
		Small nose	**-**	**-**	**-**	**-**	**+**	**+**	**+**	**-**	**-**	**+**	**-**	**-**	**-**	**+**	**-**	**-**
		Small nostrils	**-**	**-**	**+**	**-**	**+**	**+**	**+**	**-**	**-**	**+**	**+**	**+**	**-**	**+**	**+**	**+**
		“Parentheses” grooves^a^	**-**	**+**	**-**	**+**	**-**	**-**	**m**	**-**	**-**	**-**	**-**	**-**	**-**	**+**	**-**	**-**
	Commonly found in DA2A	Non-"H shaped" cutaneous dimples^b^	**-**	**+**	**-**	**+**	**+**	**-**	**-**	**-**	**-**	**+**	**-**	**-**	**-**	**-**	**+**	**-**
		Prominent supraciliary ridge ^c^	**-**	**-**	**+**	**-**	**+**	**m**	**-**	**+**	**-**	**-**	**-**	**-**	**-**	**+**	**-**	**-**
		CFCM^d^	**+**	**-**	**-**	**-**	**-**	**-**	**+**	**-**	**-**	**+**	**+**	**+**	**+**	**+**	**-**	**—**
		Dental crowding	**-**	**-**	**-**	**+**	**-**	**-**	**-**	**-**	**-**	**-**	**+**	**+**	**+**	**+**	**-**	**-**
		Pinched/protruding/pouting lips	**p**	**pll**	**po**	**pll**	**po**	**-**	**pll**	**-**	**-**	**po**	**p**	**pll**	**p**	**p**	**-**	**-**
		Hypertelorism	**+**	**+**	**-**	**-**	**+**	**-**	**+**	**-**	**-**	**-**	**-**	**+**	**-**	**-**	**-**	**-**
		Epicanthal folds	**+**	**+**	**-**	**-**	**+**	**-**	**-**	**-**	**-**	**-**	**-**	**+**	**-**	**-**	**-**	**-**
		Potosis	**-**	**-**	**M**	**-**	**-**	**-**	**-**	**+**	**-**	**+**	**-**	**-**	**-**	**-**	**m**	**-**
		Strabismus	**-**	**-**	**-**	**-**	**-**	**-**	**m**	**+**	**+**	**-**	**-**	**+**	**-**	**-**	**-**	**-**
		Very small mouth	**-**	**-**	**-**	**-**	**+**	**-**	**-**	**-**	**-**	**-**	**-**	**-**	**-**	**-**	**-**	**-**
		Notching-like of the alae	**-**	**m**	**-**	**m**	**-**	**-**	**m**	**-**	**-**	**-**	**-**	**-**	**m**	**m**	**m**	**-**
		“H-chin”^e^	**-**	**-**	**-**	**-**	**-**	**-**	**-**	**-**	**-**	**-**	**-**	**-**	**-**	**-**	**-**	**-**
	Shared in DA2A and DA2B	DS palpebral fissures	**+**	**+**	**+**	**+**	**-**	**+**	**-**	**+**	**-**	**+**	**+**	**-**	**+**	**-**	**+**	**-**
		Prominent nasolabial folds	**+**	**+**	**+**	**+**	**+**	**+**	**+**	**+**	**+**	**+**	**+**	**+**	**+**	**+**	**+**	**+**
		Long philtrum	**-**	**-**	**-**	**-**	**+**	**-**	**-**	**-**	**+**	**+**	**+**	**+**	**-**	**+**	**+**	**+**
		Attached ear lobes	**+**	**-**	**-**	**+**	**-**	**-**	**-**	**-**	**-**	**-**	**+**	**-**	**-**	**-**	**-**	**-**
		Low set ear	**-**	**-**	**-**	**-**	**+**	**-**	**-**	**-**	**-**	**+**	**-**	**+**	**-**	**-**	**-**	**+**
	Commonly found in DA2B	Micrognathia	**+**	**-**	**-**	**-**	**-**	**-**	**-**	**-**	**-**	**+**	**-**	**+**	**+**	**+**	**-**	**-**
		Small mouth	**+**	**+**	**+**	**+**	**-**	**+**	**+**	**+**	**+**	**-**	**-**	**-**	**-**	**-**	**-**	**+**
		Triangular face	**-**	**-**	**+**	**+**	**-**	**-**	**-**	**-**	**-**	**-**	**-**	**-**	**-**	**-**	**-**	**-**
		Small, pointed chin	**-**	**-**	**-**	**+**	**-**	**-**	**-**	**-**	**-**	**-**	**-**	**-**	**-**	**-**	**-**	**-**
**Limbs**	Hands	Ulnar deviation of fingers	**-**	**+**	**-**	**-**	**+**	**+**	**+**	**+**	**+**	**+**	**-**	**-**	**+**	**+**	**+**	**+**
		Adducted stiff thumb	**-**	**+**	**+**	**+**	**+**	**+**	**+** ^g^	**+**	**-**	**+**	**-**	**-**	**+**	**-**	**+**	**+**
		Clasped thumb	**-**	**+**	**-**	**-**	**-**	**-**	**-**	**-**	**+**	**+**	**-**	**-**	**+**	**+**	**-**	**-**
		Camptodactyly	**-**	**+**	**+**	**+**	**+**	**+**	**+**	**+**	**+**	**+**	**+**	**+**	**+**	**+**	**+**	**+**
		Contractures in MP joints	**-**	**+**	**-**	**-**	**-**	**-**	**-**	**-**	**+**	**+**	**-**	**-**	**-**	**-**	**-**	**+**
		Abnormal flexion creases	**-**	**+**	**+**	**+**	**+**	**+**	**+**	**+**	**+**	**+**	**+**	**+**	**+**	**+**	**+**	**+**
	Feet	Vertical talus	**-**	**-**	**-**	**-**	**+**	**-**	**-**	**-**	**-**	**-**	**-**	**-**	**-**	**-**	**-**	**-**
		Talipes varus	**-**	**-**	**r**	**-**	**-**	**-**	**-**	**-**	**-**	**+** ^i^	**-**	**-**	**-**	**-**	**-**	**+**
		Planovalgus	**-**	**+**	**l**	**l**	**-**	**-**	**l**	**-**	**+**	**-**	**-**	**-**	**-**	**-**	**-**	**-**
		Overriding toes	**-**	**+**	**+**	**+**	**-**	**+**	**+**	**+**	**+**	**+**	**+**	**-**	**-**	**-**	**-**	**-**
		Flexed toes	**-**	**+**	**+**	**+**	**m**	**+**	**+**	**+**	**-**	**+**	**+**	**-**	**-**	**-**	**-**	**+**
		Overextension contractures^f^	**-**	**-**	**-**	**+** ^h^	**-**	**-**	**+** ^h^	**+** ^h^	**+** ^h^	**-**	**-**	**-**	**-**	**-**	**-**	**+** ^h^

DA2 (A/B), distal arthrogryposis type 2(A/B); MP, metacarpophalangeal; O, oval; R, rectangular; T, triangular; S, square; D, diamond; +, present;-, absent; m, mild; p, pinched; pll, protruding lower lip; po, pouting; DS, down slanting; l, left; r, right;

^a^ Two vertical grooves that extending from the prominent nasolabial folds downward below the corners of the mouth so that they exhibit like parentheses around the mouth;

^b^ Cutaneous dimples below the lower lip on the chin or the sides of the chin;

^c^ Prominent supraciliary ridges, which is a soft, subcutaneous swelling across the lower forehead just above the eyebrows;

^d^ A crease extended laterally and downward from the corners of the mouth (abbreviated as “CFCM”);

^e^ “H chin” referred to H-shaped dimpling of the chin;

^f^ Overextension contractures, which are dorsally flexed contraction at the metatarsophalangeal joints of the toes or overextension contractures at the proximal joints of the toes

^g^ Her thumbs were adducted but not stiff.

^h^ Overextension contractures were observed at the metatarsophalangeal joints of the toes of individuals X5, X12 and H40, while at the proximal interphalangeal joints of the toes of individuals X13 and X19;

^i^ His foot deformity conformed to talipes varus after several surgical corrections of severe talipes equinovarus(clubfoot)

**Fig 3:**
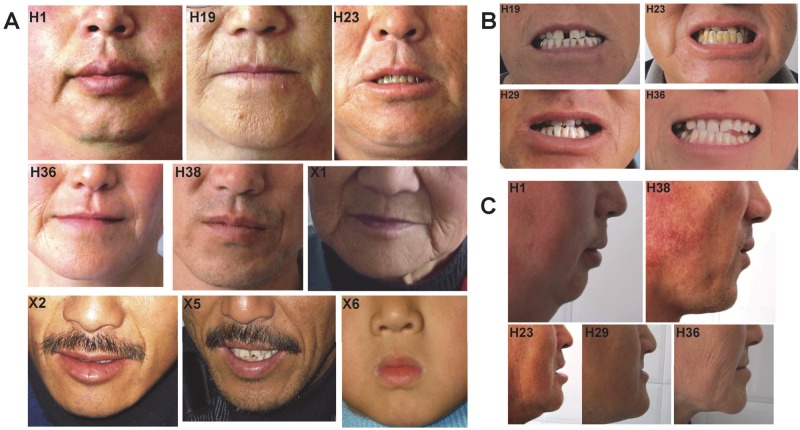
Facial contractures in affected individuals from both Chinese DA2 families. **A**: Note that prominent nasolabial folds and small nose with small nostrils were present in the affected individuals. Pouting lips (H1, H23, X2, X5 and X6) or pinched lips (H19, H36 and X1) were noticed. A crease extending laterally and downward from corners of the mouth (prominent in H1 and X1, mild in H19, H23 and H36) or “non-H-shaped” cutaneous dimples on the sides of the chin (H38, X2 and X5) were also observed. The prominent nasolabial folds extended downward below the corners of the mouth so that which exhibiting like “parentheses” around the mouth (X2 and X5). A very small whistling mouth (X6) and a deep vertical groove besides the left corner of the mouth (H38) were remarkable. **B**: Note that dental crowding were found in four affected individuals of pedigree H. **C**: Micrognathia was also present in pedigree H.

Minor facial anomalies meeting the diagnosis of DA2 including downslanting palpebral fissures, prominent nasolabial folds, long philtrum, micrognathia, small mouth and small nose with small nostrils were present in these two families. Hypertelorism, ptosis, strabismus, dental crowding and pinched/pouting lips were also observed (Table 1 and Fig. 3). Remarkably, a crease extending laterally and downward from corners of the mouth or “non-H-shaped” cutaneous dimples on the sides of the chin were also present. In several affected individuals, the prominent nasolabial folds extended downward below the corners of the mouth so that which exhibiting like “parentheses” around the mouth (Fig. 3). Prominent superciliary ridges were well defined in three individuals of family X. Moreover, two vertical grooves which paralleling with “the parentheses” around the mouth on the cheeks of case X5, a very small mouth with pouting lips of case X6 and a deep vertical groove besides the left corner of the mouth of case H38 deeply caught our eyes (Fig. 3).

Detailed neurological examinations were performed on the probands of the two families and no weakness of any specific muscle groups was noted. We could not get any muscle biopsy from our patients affected with DA2 to perform the evaluation of myofibril change. Feeding problems during the neonatal period were not reported. Physical examination showed a high degree of variability in expression, from the asymptomatic carrier to full penetrant affected individuals with severe camptodactyly and facial contractures. Blood samples were collected from 20 and 40 members of families X and H, respectively.

### *MYH3* re-sequencing

Genomic DNA was isolated for direct sequencing and linkage mapping using standard methods [11]. We first performed mutation screening in *MYH3* using previously reported primers in the probands of these two DA2 families(case X6 and case H20) because mutations in this gene are the most common known cause of distal arthrogryposis [6].

### Linkage analysis and haplotype analysis

After sequencing analysis of *MYH3*, linkage analysis was performed using 14 microsatellite markers flanking the 6 reported DAs loci (detailed in Table 2) in these two DA2 families. It seems likely that recombination events happened to 2 of the three microsatellite markers flanking the *MYBPC1* locus in family X. Then further linkage analysis using a more set of 7 markers flanking the locus was performed in this pedigree. Two-point linkage analysis was carried out using the MLINK program of the LINKAGE Package (version 5.2) with the following parameters: autosomal dominant inheritance, penetrance of 0.95, a mutation rate of zero, equal male-female recombination rate, equal microsatellite allele frequency, and a disease allele frequency of 1 in 10,000. To confirm that the DA2 phenotype was introduced into the pedigree X by the germline mosaicism of de novo *MYBPC1* mutation in case X1, haplotypes were constructed manually from the observed genotype data of the microsatellite markers.

**Table 2:** Primer sequences of the microsatellite markers used in linkage mapping.

Gene Loci	Markers	Primer Sequence(5′-3′)	Size(bp)	Annealing T(°C)
TPM2	D9S1817-F	AGCTGTAGTGAGCCCTGAT	229–277	59
	D9S1817-R	CGTTAGGAGCCTTGAGACTT		
	D9S1878-F	TCCTTGGACAAGTACCAACA	263–293	59
	D9S1878-R	GGGAAACACAGTGCCTCT		
	9chAAC-F	GGCTGAAGCAAGCAAATCAC	211	56
	9chAAC-R	CTCAGTCCCTTCACATTGTTGT		
TNNT3/TNNI2	D11S4046-F	ACTCCAGCCTGGGAAAC	183–203	59
	D11S4046-R	TGATAGACACACCCATTGC		
	11chCCT-F	CTACAAGGAAAGCTGGGTC	217	62
	11chCCT-R	AAGGGCTGGAAGAGGTGG		
TNNC3	20chAC-F	TGAGCAGGTCCAACTCT	155	56
	20chAC-R	TGTTCTGGAAGGAGGG		
	D20S888-F	GGACTTGCTAAGCCTCCAC	141–167	62
	D20S888-R	GTCAGGGCTCCCTAGAGAA		
	D20S911-F	TCCCAAGTGCCTAGAAGAG	173–187	59
	D20S911-R	GGCCCAATTTGTAGTTCAG		
MYBPC1*(panel 1)	D12S78-F	CTTTGCAGCACCATGTATTT	171–201	53
	D12S78-R	ACTGCTGGCTTTAACAGAAA		
	D12S1727-F	AGTCACCACTGAAAATCCAC	171–181	55
	D12S1727-R	GAGTGAGACCCCGTAAAAA		
	D12S1641-F	ATCAGTCAGGCAGTGTGCTA	138–150	65
	D12S1641-R	TTTAAGCCCTCATANCCTCT		
*MYBPC1* *(panel 2)	D12S332-F	GAAACTATTGGGCTGCTGA	317–341	55
	D12S332-R	AGTCGTAATTGGGAACAAAA		
	D12S1588-F	ACCGTCTGAAGAATGCG	202–218	55
	D12S1588-R	CCGAATGTTAGGGGCTG		
	D12S1041-F	AACTGTGGAAAAAGGGGAAC	140–152	55
	D12S1041-R	TGCAACAAACCACCATGG		
	D12S1607-F	AGCTGAGATCATGCCACTG	131–159	55
	D12S1607-R	TTGGTGAGCCCTGAAGAA		
	IGF1-F	GCTAGCCAGCTGGTGTTATT	176–196	55
	IGF1-R	ACCACTCTGGGAGAAGGGTA		
	D12S318-F	AGCTTCTCCACTGTGTGNTT	246–263	55
	D12S318-R	CTTGAATAANTGATATGTTACCCA		
	D12S1030-F	TCCCACATTGACCTATGTAGG	243–271	55
	D12S1030-R	AGAGTGTAAATGCTACAAGGGC		
FBN2	CAA1-F	ACCATATCACAGAGTCTCCC	173	57
	CAA1-R	TCCGTTTCATTCCTTGTCTG		
	TAA1-F	ACATGGTGAAACCCCGTCTC	162	59
	TAA1-R	ACCTCCTCCCTGCAACTTCC		
HOXD10	AT-F	AGTGATCTGTAATCCCTATG	115	53
	AT-R	ATGCGCAATAATAGTTACCC		

*The microsatellite markers of panel 1 flanking *MYBPC1* were used in linkage analysis of both families, while the panel 2 was used in a further linkage analysis of pedigree X only.

### Mutation analysis

According to mapping results, *MYBPC1* (NM_002465.3) gene was sequenced in the probands (case X6 and case H20) of the two families using previously reported primers [12]. A novel *MYBPC1* mutation was found in each proband of the families. Ecl136II recognition site was eliminated by the mutation in pedigree X, while Hinf I recognition site was introduced by the novel mutation in pedigree H. To confirm the variations, PCR was performed with primer pairs XF/XR or HF/HR (Table 3) using DNA from all family members of pedigree X or H, respectively, and the PCR product was digested with the restriction enzyme Ecl136II or Hinf I for pedigree X or H according to the protocols of manufacturers. Digested products were fractionated by 8% polyacrylamide gel electrophoresis (PAGE) and analyzed by silver staining. We used this method to screen for the mutations in 220 ethnically-matched chromosome controls. To demonstrate the somatic mosaicism of case X1 in the pedigree X does not exist in her lymphocytes, the amplification refractory mutation system (ARMS) approach was applied. Primer pairs (sequences not given but available upon request) used in ARMS-PCR were designed with online tool (http://primer1.soton.ac.uk/primer1.html)[22]. The ARMS-PCR was carried out with 2×Power Taq PCR MasterMix (BioTeke, Beijing, China). Products were detected and analyzed as RFLP analysis.

**Table 3:** Primer sequences used in RFLP analysis of *MYBPC1* in each DA2 family.

Pedigree	Primer Name	Primer Sequence(5′-3′)	Size(bp)	Annealing T(°C)
X	XF	ATGTGACAGCCGGTGATGAG	102	55
	XR	TGGGAGAAGAAGCCAACAGC		
H	HF	AAAGGATAATTTGGAAGGTTTCTG	421	55
	HR	TGTGACTTAAATGTTTACTCTCTCTCC		

RFLP, restriction fragment length polymorphism

### *In silico* prediction of protein function

The possible impact of amino acid substitution on the structure and function of human proteins was estimated with *in silico* prediction of protein function with the following tools: SIFT (http://sift.jcvi.org/) and PolyPhen-2 (http://genetics.bwh.harvard.edu/pph2/). To estimate the importance of the novel mutations, Vertebrate MultiZ Alignment and Conservation of the region surrounding each affected residue were obtained from the UCSC genome browser (http://genome.ucsc.edu/). The reference sequences used for *MYBPC1* gene with these tools were ENSP00000354849 or ENST00000361466 (Ensembl).

### Ethics statement

The individual in this manuscript has given written informed consent (as outlined in PLOS consent form) to publish these case details. Parents or legal guardians provided written informed consent on behalf of minors and this study was approved by the Institutional Review Board at the Key Laboratory of Reproductive Health of the Liaoning Province (Shenyang, China).

## Results

### Linkage of the DA2 phenotype in these families to *MYBPC1* gene locus

We first performed mutation screening in *MYH3* in these two DA2 families and no disease-causing mutations were found. Then we performed linkage analysis using 14 microsatellite markers flanking the 6 reported DAs loci (Table 2). Positive LOD scores of 3.01 or 7.22 at theta = 0 were obtained with the markers close to the *MYBPC1* gene in pedigree X or H respectively, corresponding to the DA1B locus, showing definitive evidence of linkage (Table 4). LOD scores obtained from the markers flanking the rest DAs loci excluded genetic linkage (data not shown).

**Table 4:** Two-point linkage analysis using genetic markers flanking the *MYBPC1* in these two DA2 families.

Pedigree	Memo	Marker	LOD score at						
			θ = 0.0	θ = 0.01	θ = 0.05	θ = 0.1	θ = 0.2	θ = 0.3	θ = 0.4
H		D12S1641	-infini	5.41	5.65	5.34	4.31	2.98	1.38
		D12S1641*	7.22	7.11	6.65	6.04	4.71	3.20	1.48
		D12S1727	4.80	4.73	4.46	4.08	3.21	2.20	1.05
		D12S78	-infini	-0.44	0.74	1.06	1.05	0.72	0.27
X	Crossover	D12S1641	-infini	1.26	1.77	1.81	1.55	1.09	0.53
	Crossover	D12S332	-infini	-0.51	0.12	0.32	0.42	0.36	0.22
	Crossover	D12S1588	-infini	-3.32	-1.35	-0.61	-0.06	0.07	0.04
		D12S1041	1.75	1.70	1.53	1.33	0.94	0.58	0.24
		D12S1727	1.26	1.23	1.09	0.93	0.63	0.35	0.12
		D12S1607	3.01	2.96	2.74	2.47	1.87	1.23	0.56
		IGF1	1.51	1.48	1.39	1.28	1.02	0.73	0.40
		D12S318	2.80	2.74	2.51	2.21	1.58	0.91	0.31
		D12S1030	2.71	2.67	2.49	2.25	1.74	1.16	0.53
	Crossover	D12S78	-infini	-2.43	-0.51	0.16	0.57	0.57	0.36

*Removed individual H25 (asymptomatic carrier) in calculation of LOD score

### Identification of two novel mutations in the *MYBPC1* gene

Subsequent sequencing of *MYBPC1* in the probands of the two families identified two novel missense mutations, c.1075G>A (p.E359K) in pedigree X and c.956C>T (p.P319L) in pedigree H (Fig. 1). Through polymerase chain reaction (PCR) using specific primer pairs (Table 3) followed by RFLP analysis, each of the mutations was present in all affected family members and the asymptomatic carrier, and absent in unaffected family members of pedigree X or H (Fig. 1). Both mutations were not identified in 220 control chromosomes of Han Chinese ancestry. Both of the substitutions occur within C2 immunoglobulin domain, which together with C1 and the M motif constitute the binding site for the S2 subfragment of myosin (Fig. 1). Additionally, these two variants were not found in LOVD (http://www.lovd.nl/3.0/home), dbSNP (build 132, http://www.ncbi.nlm.nih.gov/projects/SNP/ and the 1000 Genomes Project pilot data (http://browser.1000genomes.org/index.html). The results of ARMS-PCR analysis in pedigree X were consistent with RFLP analysis in this family and the PAGE pattern (data not shown) was similar to that of RFLP analysis (Fig. 1). Somatic mosaicism was not detected in her lymphocytes of case X1 in pedigree X and the other somatic samples such as buccal cells, urine sediment, vaginal, and/or cervical cells were not available for the detection of somatic mosaicism.

### Prediction of protein function for the two MYBPC1 substitutions

Protein sequence alignment of MYBPC1 orthologs showed that the residues of the two variants are highly conserved down to zebrafish (Fig. 1). Similar to the previously reported DA1 mutations in *MYBPC1*, assessment using SIFT and PolyPhen-2 predicted tolerated and damaging effects due to these DA2 mutations (Table 5). We believe those novel substitutions (p.E359K and p.P319L) in MYBPC1 are the pathogenic mutations in the two Chinese families with DA2.

**Table 5:** Predicted effects due to the mutations in *MYBPC1* using SIFT and PolyPhen-2.

Variants	Domain	SIFT	PolyPhen-2	Memo
p.P319L	C2	Tolerated(0.29)	Possibly damaging(0.593)	reported in the present study
p.E359K	C2	Tolerated(0.11)	Probably damaging(0.999)	reported in the present study
p.W236R	M motif	Tolerated(0.43)	Probably damaging(0.999)	[12]
p.Y856H	C8	Tolerated(0.31)	Probably damaging(0.997)	[12]

SIFT: scores % less than 0.05 indicate substitutions are predicted as intolerant.

PolyPhen-2: scores are evaluated as 0.000 (most probably benign) to 0.999 (most probably damaging).

## Discussion

### Classification of these two DA2 families and distinctive facial and limb features

DA is a group of clinically and genetically heterogeneous disorders. Classification of DAs may be difficult due to reduced penetrance, variable expressivity and overlapping features of different forms, particularly among DA1, DA2B and DA2A. In a recent report, DA1 and DA2B were suggested be phenotypic extremes of the same disorder [4]. Even in some studies DA1, DA2B and DA2A were proposed in a phenotypic continuum of the same disorder [23,24]. The most recent report’s findings indicate that DA3 and DA5 are etiologically related and perhaps represent variable expressivity of the same condition [18]. Despite the recognition of 2 distinct syndromes, DA2A (FSS) and DA2B (SHS), differential diagnosis between these 2 disorders is so challenging that a strict diagnostic criteria for classical FSS was proposed [5]. Except distal contractures in our two DA2 families (Fig. 2 and Table 1), substantial facial findings meeting the diagnosis of DA2 were observed (Table 1 and Fig. 3). As a result, the two Chinese families evaluated in this study were diagnosed with DA2. However, it’s too challenging for us to fit them accurately into DA2A/FSS or DA2B/SHS. In the opinion of Professor Michael Bamshad, “there is no a typical H-chin from our affected individuals, and there are a few dimplings of the lower lip on several chins from our families, however, they are not more specific to that of FSS”(personal communication with Professor Michael Bamshad). Thus the clinical findings of these DA2 families seem to be more consistent with DA2B even though there is no triangular face with small pointed chin in most affected individuals (Table 1 and Fig. 3). However, a crease extending laterally and downward from the corners of the mouth was found in 2 of 9 patients in pedigree X and 5 of 7 in pedigree H, respectively. This feature was first described by Fraser, et al. in an adult with FSS [25] and also found in the most reported FSS adults (Group 1 in Table 6). Moreover, this feature was remarkable in most of the reported infants or children with FSS of classical H-chin (Group 2 in Table 6). More attractively, in some reports the features of the chin of the individuals with FSS could be described as “H-chin”, as well as “a crease extending laterally and downward from the corners of the mouth”(Group 3 in Table 6). In contrast, this feature was absent in most reported individuals with DA2B (Group 4 in Table 6). Additionally, other commonly found features in FSS including dental crowding, pininched/pouting lips, strabismus and very small mouth were also observed in these two DA2 families (Table 1and Fig. 3). Thus we could not exclude the possibility that these two DA2 families are affected with DA2A. Remarkably, in three affected individuals of pedigree X the prominent nasolabial folds extended downward below the corners of the mouth so that which exhibiting like “parentheses” around the mouth. Two vertical grooves which paralleling with “the parentheses” were present on the cheeks of case X5 (this feature was also found in another two DA2 patients of our other 2 DA pedigrees. Unpublished data) and a deep vertical groove besides the left corner of the mouth of case H38 also deeply caught our eyes (Fig. 3). The deep vertical groove besides the corner of the mouth and the “prentheses” were not previously reported and are more likely to be the residual form of “dimpling of the lips”.

**Table 6:** Summary of the features of the chin of FSS or SHS cases from literatures.

Features of the chin	Cases and Figures	Reference	Memo
A crease extending laterally and downward from the corners of the mouth (abbreviated as “CFCM” below) was present in the reported limited number of adult FSS cases.	Case 5, Fig 5	[25]	Group 1
	Case 2, Fig 3	[39]	
	Case II8, Fig 2	[40]	
	Case 2, Fig 8 right	[41]	
	Case 2, Fig 3	[7]	
	Case 1, Fig 1	[42]	
	Case father in Fig 4 and Case of panel 2 in Fig 5	[5]	
	Cases II2, I2 and III1 in supplementary Fig. S2	[23]	
The “CFCM” was also remarkable in most of the reported infants or children with FSS of classical “H shaped dimpling of the chin”(H chin).	Case Renate K., Fig 1	[43]	Group 2
	Case 1, Fig 1	[44]	
	Case of kulz, center left in Fig 1	[45]	
	Patient 2, Fig 2	[46]	
	Case 1, Fig 1	[39]	
	Cases top left 2 and bottom left 2 in Fig 2	[5]	
	Case a, Fig 1	[6]	
	Case 1, Figs 1–4	[47]	
	Case 1, Figs 1 and 2	[48]	
The features of the chin of FSS cases could be described as either “H chin” or “the CFCM”.	Case 2, Fig 3	[39]	Group 3
	Case 2, Fig 3	[7]	
	Case 1, Fig 1	[42]	
	Case 1, Figs 1	[49]	
	Cases top center, Fig 2	[5]	
	Case 1, Fig 2	[50]	
The feature of “CFCM” was absent in most of the reported individuals with DA2B.	Cases 1, 2 and 3 (unpublished data)	[11]	Group 4
	Cases III-15 and IV-12 in Fig 2; Cases I-1 and III-4 in Fig 4	[51]	
	Individual V-5 in Fig 2; Individuals I-2 and II-1 in Fig 4	[52]	
	Index case in Fig 1	[53]	

Except DA10 characterized by plantar flexion contractures could be recognized from other DA syndromes by the distinctive lower limb phenotype[26], other forms of DA may have such similar limb phenotypes that they can hardly be distinguished from one another based on distal limb contractures alone [27]. However, intrafamilial variability of the penetrance of hand contractures was underscored to be considerable in families with DA1 [12]. In their DA1B families with *MYBPC1* mutations, the lower limb contractures were full penetrant and more severely affected than the upper limbs [12]. In contrast, the upper limb contranctures were more penetrant and severely affected than the lower limbs in these DA2 families caused by *MYBPC1* mutations (details in Table 1). Although the same type mutations in the same gene located in different domains leading to different types of DA have been exemplified by missense mutations in *MYH3*, almost all FSS mutations are predicted to affect ATP binding and hydrolysis domain of embryonic myosin whereas mutations that cause SHS disturb amino acid residues on the surface of embryonic myosin[6]. Due to the limited DA cases with *MYBPC1* mutations reported, we could not draw a positive correlation between genotype and phenotype similar to that of *MYH3* mutations causing FSS or SHS. And thus it seems not clear whether the limbs phenotypic difference between the DA1B and these DA2 families resulted from each own genotype or other factors. On the other hand, together with the study of DA1B families caused by *MYBPC1* mutations, our reports of *MYBPC1* mutations in DA2 families may add further genetic evidence supporting the hypothesis that DA1, DA2B and DA2A may be in a phenotypic continuum of the same disorder. The overextension contracture was only reported in the first DA1B family and it was present at the proximal interphalangeal joint of the fourth finger in two individuals [12]. Notably, overextension contractures were also observed in these two Chinese DA2 families caused by *MYBPC1* mutations. However, these overextension contractures were observed at the metatarsophalangeal joints or the proximal interphalangeal joints of the toes of the affected individuals (Fig. 2 and Table 1). More studies are needed to ascertain whether overextension contracture is specific to the DA patients with *MYBPC1* mutations.

### Germline with somatic mosaicism in case X1 of pedigree X

**Fig 4:**
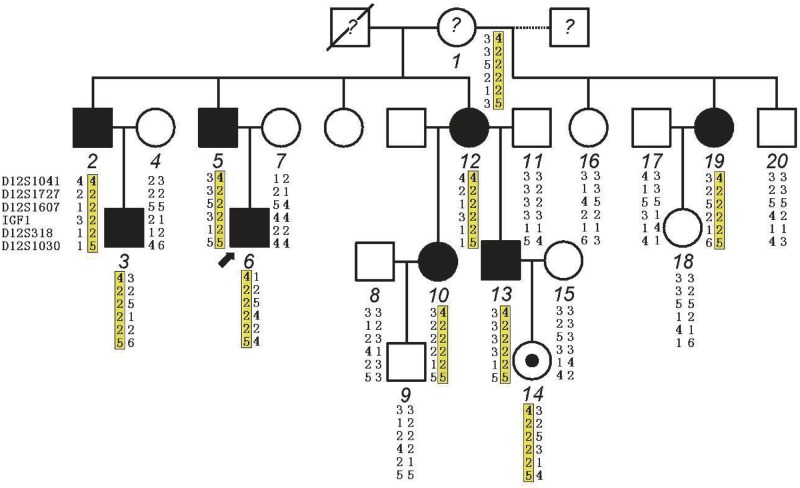
Haplotype analysis in pedigree X. Haplotype analysis indicating microsatellite markers on chromosome 12q flanking *MYBPC1* common to all affected individuals with distal arthrogryposis type 2. Common haplotype is highlighted in yellow and derived from the mildly symptomatic founder (case X1). The question mark indicates the unknown status of the individuals of family X. Case X1 had minor facial anomalies, suggesting she may have a somatic mosaicism of the MYBPC1 mutation (p.E359K). The small filled circle inside the open circle indicates an asymptomatic carrier. Marker’s name is shown at the left. Dotted line in pedigree X indicates individuals 16, 19 and 20 were born from different fathers.

Mosaicism in germ cells has been recognized as an important and relatively frequent mechanism at the origin of genetic disorders[28]. Some pedigrees with DAs introduced by germline mosaicism (GM) of *TNNI2* or *TNNT3* mutations have been reported [4,9,23]. In pedigree X, case X1 was first taken as clinically normal individual. Haplotype analysis indicated common haplotype shared by all affected individuals is derived from case X1 (Fig. 4). This finding suggests that she may be an asymptomatic carrier. However, the *MYBPC1* mutation p.E359K in this pedigree was not detected in her lymphocytes by direct sequencing, RFLP analysis and ARMS-PCR approaches. These findings suggest that DA2 of pedigree X may be introduced by GM which exists in her germ cells. The founder of kindred X, case X1 was examined for clinical evaluation again. Minor facial contractures were noted (Table 1 and Fig. 3) and both her upper and lower limbs were not affected indicating that she is a mildly symptomatic individual and may also have the somatic mosaicism of the *MYBPC1* mutation. Although it was not detected in her lymphocytes, it may exist in cell populations that were not tested (e.g., buccal cells, urine sediment, vaginal, or cervical cells). Germline mosaicism violates the assumptions underlying classic genetic analysis and may lead to failure of such analysis. Fortunately, common haplotype of the affected individuals was not shared by the unaffected individuals (X16 and X20) in pedigree X (Fig. 4) so that solid statistical evidence could be obtained from classical two point linkage analysis in this kindred. Otherwise, the extended the statistical model used for genetic linkage analysis in the presence of germline mosaicism [29] or the whole exome sequencing could be introduced in identification of disease-causing gene. This is the first report of one big DA2 pedigree introduced by germline mosaicism of the de novo *MYBPC1* mutation.

### Function prediction

**A possible mutation hot spot at the NH2-terminus of MyBP-C slow**. To date, only three *MYBPC1* mutations have been reported in human disease. Except the two distinct DA1B missense mutations[12], a homozygous *MYBPC1* nonsense mutation was reported recently in autosomal recessive lethal congenital contracture syndrome type 4(LCCS4)[21]. The DA1 MYBPC1 p.W236R and p.Y856H mutations are located within the M motif and the C-terminal C8 domain, respectively [12], while both the LCCS4 (p.R318X) mutation and our two DA2 (p.P319L and p.E359K) mutations in MYBPC1 are located in the C2 domain (Fig. 1). It seems likely that the fragment of M motif and C2 domain is a mutation hot spot (4/5) at the NH2-terminus of MyBP-C slow. Nevertheless, this finding was drawn from the limited reports and more *MYBPC1* mutations involved in human disease are needed.

**Impact on energy metabolism and homoeostasis during muscle contraction**. Muscle contraction requires high-energy fluxes that are supplied by muscle-type creatine kinase (MM-CK), which couples to the myofibril. This coupling is mediated by MYBPC1: MM-CK binds to the C-terminal domain of MYBPC1, which is also the binding site of myosin. Thus, MYBPC1 acts as an adaptor connecting the ATP consumer (myosin) and the regenerator (MM-CK) for efficient energy metabolism and homoeostasis [30]. Given that the coupling of MM-CK to the myofibril by MYBPC1 through its C-terminal fragments (domains C6–C10), but not its NH2-terminal fragments[30], thus the crucial role of MYBPC1 in energy homoeostasis during muscle contraction does not seem to be affected by our two DA2 mutations and one reported DA1 mutation (p.W236R), located within C2 domain and M motif, respectively. On the contrary, the other DA1 mutation (p.Y856H) was located within C8 domain, which was included in the COOH-terminal mediate fragment of MYBPC1 for recruitment of MM-CK to myosin and therefore the adaptor role of MYBPC1 to bridge MM-CK and myosin seems likely to be impacted. It seems possible that the imbalance between ATP production and utilization in muscle contraction may also underlie the pathogenesis of DA1 myopathy associated with the p.Y856H mutation.

**Important functional role of domain C2 of MYBPC1 and possible pathogenic mechanism**. Much of our knowledge on ligands of MyBP-C originates from the numerous studies that focus on the cardiac isoform, binding of the skeletal isoforms of MyBP-C with their partners is much less characterized [19,31]. Further characterization of the NH2-terminal binding of slow MyBP-C has merely shown that the first two immunoglobulin domains (C1–C2) bind to the S2 region of myosin [32], while the contribution of each domain (C1, M motif and C2) in this binding is still elusive. On the contrary, binding site of each domain of the cardiac MyBP-C on S2 region has been well recognized [19]. Specifically, the M motif has been shown to bind directly to the NH2-terminal 126 residues of the S2 fragment (S2Δ) [33]. Similarly, the C2 domain has been also shown to interact with the same S2Δ fragment, albeit with considerably lower affinity but a highly specific binding, compared to the M motif[34]. Similar to its cardiac isoform, the key contributions of M motif and domain C2 of slow MyBP-C may exist in its NH2-terminal binding to S2 region of myosin. Therefore, these two DA2 *MYBPC1* mutations (p.P319L and p.E359K) in the present study may suggest the important role of domain C2 of MYBPC1 in binding to S2 fragment of myosin.

Mutations in *MYBPC3* causing autosomal dominant hypertrophic cardiomyopathy through haploinsufficiency [35] may lead us to suppose that mutations in *MYBPC1*, similar to its cardiac counterpart, cause disease through gene haploinsufficiency. However, MYBPC1 haploinsufficiency was not reported to cause muscle disease in the heterozygous carriers of the Bedoin kindreds with autosomal recessive LCCS4 due to homozygous *MYBPC1* nonsense mutations [21]. Moreover, the presence of the DA1 missense mutations does not seem to affect the expression levels of *MYBPC1* [36]. More importantly, dominant negative effects of human DA1 *MYBPC1* missense mutations on muscle function have been demonstrated in zerbrafish models of arthrogryposis[37]. Thus, it is likely reasonable to speculate that these two DA2 families associated with p.E359K and p.P319L mutations may be caused by dominant negative impairment on the regulatory properties of the NH2 terminus of MYBPC1.

### Possible molecular mechanisms

Although, there are extensive studies on domain mapping of the cardiac MyBP-C in binding with its partners [19]; Exogenous expression of *MYBPC1* containing the human DA1 mutations in murine muscle demonstrated correct sarcomeric localization of MYBPC1 mutant proteins [12]; In vitro binding and motility assays showed that the actomyosin regulatory properties of MYBPC1 are completely abolished by the presence of the DA1 mutations [38]; and dominant-negative effects of human DA1 *MYBPC1* missense mutations have been suggested in zebrafish models of arthrogryposis[37], the molecular mechanism that may underlie the pathogenesis of DA2 myopathy associated with the p.P319L and p.E359K mutations is currently under investigation. The structure of domain C2 of human cardiac MyBP-C (cC2) by NMR spectroscopy and a realistic structural model of the cC2–S2Δ complex have been proposed [34]. Domain cC2 has the β-sandwich structure expected from a member of the immunoglobulin I-set. One sheet is formed by strands ABED, and the other by strands C‘CFGA’. According to the alignment of sequences of domain C2 from various MyBP-C isoforms [34], our DA2 mutations, p.P319L and p.E359K, are located in the linker between strands C’ and D and the C terminal strand G, respectively. And strand G was included in the C‘CFGA’ β-sheet on which the S2Δ specific binding site was located. In their structural model, cC2 alone binds to S2Δ with low affinity but exhibits a highly specific binding site through the surface charge complementarities which resulted from key polar amino acids of both proteins[34]. For cC2, Glu296 (according to the author’s numbering scheme) was included in the residues that defined the S2 binding site. Moreover, Glu296 was one of the most amino acids that make hydrogen bond and/or salt bridge interactions with adjacent residues in S2Δ. And multiple alignments revealed that this residue is extremely well conserved in C2 of all isoforms and species [34]. More significantly, our DA2 mutation p.E359K in MYBPC1 found in pedigree X corresponds to this key residue Glu296. In contrast to other IgI domains from MyBP-C there are no isoform specific insertions or deletions, so that the overall shape of the C2 domain is expected to be very similar in all isoforms [34]. Accordingly, it seems reasonable to speculate that the domain C2 of human slow MyBP-C (sC2) may interact with S2 fragment of myosin similar to cC2. Therefore, glutamic acid-359 contributes directly to the binding of the sC2 to S2 fragment of myosin. Since the absence of positive charges in the S2 binding site of cC2 is well conserved in C2 of all isoforms and species [34], thus the substitution of the negatively charged glutamic acid-359 by a positively charged lysine may directly induce an unfavorable electrostatic potential change that impact binding of sC2 to S2 of myosin, leading to loss of regulation. Consequently, the report of MYBPC1 mutation p.E359K in DA2 seems to provide further genetic evidence in its slow skeletal counterpart that support the proposed structural model of the cC2–S2Δ complex. While the other MYBPC1 mutation p.P319L in DA2 seems to be relatively far from the negatively charged S2Δ binding site and additionally both residues involved in the substitution are nonpolar and hydrophobic. Therefore, it seems likely that a different molecular mechanism may underlie the pathogenesis associated with the substitution of p.P319L, compared to p.E359K. The linker between strands C’ and D, within which the substitution p.P319L was located, lies at the C-terminal thin edge of the C2 domain with a wedge-like appearance. And the C terminus of the overall structure of cC2 makes the main contact with S2Δ in their model of cC2–S2Δ complex [34]. In addition, the distinctive cyclic structure of proline’s side chain locks its φ backbone dihedral angle at a fixed degree, giving proline an exceptional conformational rigidity compared to other amino acids. This distinct side chain/amine group interaction allows proline to play an important role in the formation of beta turn and other common turns. Although the linker between strands C’ and D was not a defined beta turn, it does be a turn from strand C’ to D, in the formation of which the proline-319 in human MYBPC1 may aid much. Accordingly, it is tempting to speculate that substitution of the distinctive proline-319 with cyclic side chain by a linear branched-chain leucine may induce an unfavorable conformational change that affects the binding of sC2 to S2 fragment of myosin.

## Conclusion

In summary, we identified two novel *MYBPC1* mutations in two large Han Chinese families with distal arthrogryposis type 2. We also observed some unique overextension contractures of the lower limbs and some distinctive facial features in our DA2 pedigrees. Our work represents the first report on the link between MYBPC1 and the DA2 phenotype, of which one was introduced by germline mosacism. Our results expand the phenotypic spectrum of MYBPC1-related arthrogryposis multiplex congenita (AMC) and we speculate that the domain C2 of MYBPC1 may play an important role in binding to S2 fragment of myosin. The substitution p.E359K in DA2 may also support the proposed structural model of the cC2–S2Δ complex and that most key interactions of the two partners are between polar amino acids. We expect this report of two novel mutations (p.P319L and p.E359K) located in C2 domain of MYBPC1 in DA2 patients and our suggestion on the possible molecular mechanisms that may underlie the pathogenesis of DA2 myopathy associated with these two substitutions will stimulate future research to further refine the details of the NH2-teminal interaction of slow MyBP-C with myosin or its other parterners and their importance for myopathy associated with AMC.

## References

[1] Bamshad M, Jorde LB, Carey JC (1996) A revised and extended classification of the distal arthrogryposes. Am J Med Genet 65: 277–281. 8923935 10.1002/(SICI)1096-8628(19961111)65:4<277::AID-AJMG6>3.0.CO;2-M

[2] Hall JG, Reed SD, Greene G (1982) The distal arthrogryposes: delineation of new entities—review and nosologic discussion. Am J Med Genet 11: 185–239. 7039311 10.1002/ajmg.1320110208

[3] Dieterich K, Quijano-Roy S, Monnier N, Zhou J, Faure J, et al (2013) The neuronal endopeptidase ECEL1 is associated with a distinct form of recessive distal arthrogryposis. Hum Mol Genet 22: 1483–1492. 10.1093/hmg/dds514 23236030

[4] Beck AE, McMillin MJ, Gildersleeve HI, Kezele PR, Shively KM, et al (2013) Spectrum of mutations that cause distal arthrogryposis types 1 and 2B. Am J Med Genet A 161A: 550–555. 10.1002/ajmg.a.35809 23401156 PMC3581718

[5] Stevenson DA, Carey JC, Palumbos J, Rutherford A, Dolcourt J, et al (2006) Clinical characteristics and natural history of Freeman-Sheldon syndrome. Pediatrics 117: 754–762. 16510655 10.1542/peds.2005-1219

[6] Toydemir RM, Rutherford A, Whitby FG, Jorde LB, Carey JC, et al (2006) Mutations in embryonic myosin heavy chain (MYH3) cause Freeman-Sheldon syndrome and Sheldon-Hall syndrome. Nat Genet 38: 561–565. 16642020 10.1038/ng1775

[7] Vanek J, Janda J, Amblerova V, Losan F (1986) Freeman-Sheldon syndrome: a disorder of congenital myopathic origin? J Med Genet 23: 231–236. 3723551 10.1136/jmg.23.3.231 PMC1049633

[8] Tajsharghi H, Kimber E, Holmgren D, Tulinius M, Oldfors A (2007) Distal arthrogryposis and muscle weakness associated with a beta-tropomyosin mutation. Neurology 68: 772–775. 17339586 10.1212/01.wnl.0000256339.40667.fb

[9] Sung SS, Brassington AM, Grannatt K, Rutherford A, Whitby FG, et al (2003) Mutations in genes encoding fast-twitch contractile proteins cause distal arthrogryposis syndromes. Am J Hum Genet 72: 681–690. 12592607 10.1086/368294 PMC1180243

[10] Sung SS, Brassington AM, Krakowiak PA, Carey JC, Jorde LB, et al (2003) Mutations in TNNT3 cause multiple congenital contractures: a second locus for distal arthrogryposis type 2B. Am J Hum Genet 73: 212–214. 12865991 10.1086/376418 PMC1180583

[11] Jiang M, Zhao X, Han W, Bian C, Li X, et al (2006) A novel deletion in TNNI2 causes distal arthrogryposis in a large Chinese family with marked variability of expression. Hum Genet 120: 238–242. 16802141 10.1007/s00439-006-0183-4

[12] Gurnett CA, Desruisseau DM, McCall K, Choi R, Meyer ZI, et al (2010) Myosin binding protein C1: a novel gene for autosomal dominant distal arthrogryposis type 1. Hum Mol Genet 19: 1165–1173. 10.1093/hmg/ddp587 20045868 PMC2838534

[13] McMillin MJ, Below JE, Shively KM, Beck AE, Gildersleeve HI, et al (2013) Mutations in ECEL1 cause distal arthrogryposis type 5D. Am J Hum Genet 92: 150–156. 10.1016/j.ajhg.2012.11.014 23261301 PMC3542461

[14] Shaaban S, Duzcan F, Yildirim C, Chan WM, Andrews C, et al (2013) Expanding the phenotypic spectrum of ECEL1-related congenital contracture syndromes. Clin Genet. 10.1111/cge.12212 23808592 PMC3883930

[15] Shaheen R, Al-Owain M, Khan A, Zaki M, Hossni H, et al (2013) Identification of three novel ECEL1 mutations in three families with distal arthrogryposis type 5D. Clin Genet. 10.1111/cge.12212 23829171

[16] Coste B, Mathur J, Schmidt M, Earley TJ, Ranade S, et al (2010) Piezo1 and Piezo2 are essential components of distinct mechanically activated cation channels. Science 330: 55–60. 10.1126/science.1193270 20813920 PMC3062430

[17] Coste B, Houge G, Murray MF, Stitziel N, Bandell M, et al (2013) Gain-of-function mutations in the mechanically activated ion channel PIEZO2 cause a subtype of Distal Arthrogryposis. Proc Natl Acad Sci U S A 110: 4667–4672. 10.1073/pnas.1221400110 23487782 PMC3607045

[18] McMillin MJ, Beck AE, Chong JX, Shively KM, Buckingham KJ, et al (2014) Mutations in PIEZO2 Cause Gordon Syndrome, Marden-Walker Syndrome, and Distal Arthrogryposis Type 5. Am J Hum Genet. 10.1016/j.ajhg.2014.12.009 24726473 PMC4067551

[19] Ackermann MA, Kontrogianni-Konstantopoulos A (2011) Myosin binding protein-C: a regulator of actomyosin interaction in striated muscle. J Biomed Biotechnol 2011: 636403 10.1155/2011/636403 22028592 PMC3196898

[20] Ackermann MA, Kontrogianni-Konstantopoulos A (2010) Myosin binding protein-C slow: an intricate subfamily of proteins. J Biomed Biotechnol 2010: 652065 10.1155/2010/652065 20396395 PMC2852610

[21] Markus B, Narkis G, Landau D, Birk RZ, Cohen I, et al (2012) Autosomal recessive lethal congenital contractural syndrome type 4 (LCCS4) caused by a mutation in MYBPC1. Hum Mutat. 10.1002/humu.22247 22610851

[22] Medrano RF, de Oliveira CA (2014) Guidelines for the tetra-primer ARMS-PCR technique development. Mol Biotechnol 56: 599–608. 10.1007/s12033-014-9734-4 24519268

[23] Li X, Jiang M, Han W, Zhao N, Liu W, et al (2013) A novel TNNI2 mutation causes Freeman-Sheldon syndrome in a Chinese family with an affected adult with only facial contractures. Gene 527: 630–635. 10.1016/j.gene.2013.06.082 23850728

[24] Kimber E, Tajsharghi H, Kroksmark AK, Oldfors A, Tulinius M (2012) Distal arthrogryposis: clinical and genetic findings. Acta Paediatr 101: 877–887. 10.1111/j.1651-2227.2012.02708.x 22519952

[25] Fraser FC, Pashayan H, Kadish ME (1970) Cranio-carpo-tarsal dysplasia. Report of a case in father and son. JAMA 211: 1374–1376. 5467037 10.1001/jama.211.8.1374

[26] Stevenson DA, Swoboda KJ, Sanders RK, Bamshad M (2006) A new distal arthrogryposis syndrome characterized by plantar flexion contractures. Am J Med Genet A 140: 2797–2801. 17103435 10.1002/ajmg.a.31528 PMC3244115

[27] Bamshad M, Van Heest AE, Pleasure D (2009) Arthrogryposis: a review and update. J Bone Joint Surg Am 91 Suppl 4: 40–46. 10.2106/JBJS.I.00281 19571066 PMC2698792

[28] Zlotogora J (1998) Germ line mosaicism. Hum Genet 102: 381–386. 9600231 10.1007/s004390050708

[29] Weissbrod O, Geiger D (2011) Genetic linkage analysis in the presence of germline mosaicism. Stat Appl Genet Mol Biol 10 10.2202/1544-6115.1666 23089820 PMC3215430

[30] Chen Z, Zhao TJ, Li J, Gao YS, Meng FG, et al (2011) Slow skeletal muscle myosin-binding protein-C (MyBPC1) mediates recruitment of muscle-type creatine kinase (CK) to myosin. Biochem J 436: 437–445. 10.1042/BJ20102007 21426302

[31] Kontrogianni-Konstantopoulos A, Ackermann MA, Bowman AL, Yap SV, Bloch RJ (2009) Muscle giants: molecular scaffolds in sarcomerogenesis. Physiol Rev 89: 1217–1267. 10.1152/physrev.00017.2009 19789381 PMC3076733

[32] Gruen M, Gautel M (1999) Mutations in beta-myosin S2 that cause familial hypertrophic cardiomyopathy (FHC) abolish the interaction with the regulatory domain of myosin-binding protein-C. J Mol Biol 286: 933–949. 10024460 10.1006/jmbi.1998.2522

[33] Sadayappan S, Gulick J, Klevitsky R, Lorenz JN, Sargent M, et al (2009) Cardiac myosin binding protein-C phosphorylation in a {beta}-myosin heavy chain background. Circulation 119: 1253–1262. 10.1161/CIRCULATIONAHA.108.798983 19237661 PMC2656413

[34] Ababou A, Gautel M, Pfuhl M (2007) Dissecting the N-terminal myosin binding site of human cardiac myosin-binding protein C. Structure and myosin binding of domain C2. J Biol Chem 282: 9204–9215. 17192269 10.1074/jbc.M610899200

[35] Marston S, Copeland O, Gehmlich K, Schlossarek S, Carrier L (2012) How do MYBPC3 mutations cause hypertrophic cardiomyopathy? J Muscle Res Cell Motil 33: 75–80. 10.1007/s10974-011-9268-3 22057632

[36] Vydyanath A, Gurnett CA, Marston S, Luther PK (2012) Axial distribution of myosin binding protein-C is unaffected by mutations in human cardiac and skeletal muscle. J Muscle Res Cell Motil 33: 61–74. 10.1007/s10974-012-9286-9 22415774 PMC3351610

[37] Ha K, Buchan JG, Alvarado DM, McCall K, Vydyanath A, et al (2013) MYBPC1 mutations impair skeletal muscle function in zebrafish models of arthrogryposis. Hum Mol Genet. 10.1093/hmg/ddt644 23873045 PMC3836476

[38] Ackermann MA, Patel PD, Valenti J, Takagi Y, Homsher E, et al (2013) Loss of actomyosin regulation in distal arthrogryposis myopathy due to mutant myosin binding protein-C slow. FASEB J. 10.1096/fj.13-240390 23657818 PMC3714579

[39] Pfeiffer RA, Ammermann M, Baisch C, Bollhoff G (1972) [Freeman-Sheldon syndrome. Three new observations]. Z Kinderheilkd 112: 43–53. 5019025

[40] Wettstein A, Buchinger G, Braun A, von Bazan UB (1980) A family with whistling-face-syndrome. Hum Genet 55: 177–189. 7450762 10.1007/BF00291765

[41] Fitzsimmons JS, Zaldua V, Chrispin AR (1984) Genetic heterogeneity in the Freeman-Sheldon syndrome: two adults with probable autosomal recessive inheritance. J Med Genet 21: 364–368. 6502650 10.1136/jmg.21.5.364 PMC1049318

[42] Lai MM, Tettenborn MA, Hall JG, Smith LJ, Berry AC (1991) A new form of autosomal dominant arthrogryposis. J Med Genet 28: 701–703. 1941966 10.1136/jmg.28.10.701 PMC1017058

[43] Otto FM (1953) [Freeman and Sheldon’s cranio-carpo-tarsal dystrophy; a casuistic report]. Z Kinderheilkd 73: 240–250. 13103378 10.1007/BF00436029

[44] Rintala AE (1968) Freeman-Sheldon’s syndrome, cranio-carpo-tarsal dystrophy. Acta Paediatr Scand 57: 553–556. 5706373 10.1111/j.1651-2227.1968.tb06979.x

[45] Weinstein S, Gorlin RJ (1969) Cranio-carop-tarsal dysplasia or the whistling face syndrome. I. Clinical considerations. Am J Dis Child 117: 427–433. 4975238 10.1001/archpedi.1969.02100030429007

[46] Cervenka J, Gorlin RJ, Figalova P, Farkasova J (1970) Craniocarpotarsal dysplasia or whistling face syndrome. Arch Otolaryngol 91: 183–187. 5410086 10.1001/archotol.1970.00770040253016

[47] Alberto Campos Molina, Verónica Sánchez Pozos, Benito Díaz Ávila, Sotelo YA (2009) Craneocarpotarsal with whistling facies syndrome Report of a case and review of the literature. 5: 103–106.

[48] SH S, S. S (2009) FREEMAN-SHELDON SYNDROME: A CASE REPORT. Iran J Child Neurology 51–54.

[49] Aren G, Yurdabakan Z, Ozcan I (2003) Freeman-Sheldon syndrome: a case report. Quintessence Int 34: 307–310. 12731619

[50] Hegde SS, Shetty MS, Rama Murthy BS (2010) Freeman-Sheldon syndrome—prenatal and postnatal diagnosis. Indian J Pediatr 77: 196–197. 10.1007/s12098-009-0227-6 20012803

[51] Krakowiak PA, Bohnsack JF, Carey JC, Bamshad M (1998) Clinical analysis of a variant of Freeman-Sheldon syndrome (DA2B). Am J Med Genet 76: 93–98. 9508073 10.1002/(sici)1096-8628(19980226)76:1<93::aid-ajmg17>3.0.co;2-k

[52] Krakowiak PA, O’Quinn JR, Bohnsack JF, Watkins WS, Carey JC, et al (1997) A variant of Freeman-Sheldon syndrome maps to 11p15.5-pter. Am J Hum Genet 60: 426–432. 9012416 PMC1712403

[53] Toydemir RM, Bamshad MJ (2009) Sheldon-Hall syndrome. Orphanet J Rare Dis 4: 11–15. 10.1186/1750-1172-4-11 19309503 PMC2663550

