# Correction: Two Novel Mutations in Myosin Binding Protein C Slow Causing Distal Arthrogryposis Type 2 in Two Large Han Chinese Families May Suggest Important Functional Role of Immunoglobulin Domain C2

**DOI:** 10.1371/journal.pone.0125310

**Published:** 2015-05-07

**Authors:** The PLOS ONE Staff

In Table 6, the figure names in the column “Cases and Figures” should not be hyperlinks. The figure names in this column are citations to figures within the references listed in the adjacent “Reference” column. Please see the corrected Table 6 below. The publisher apologizes for this error.

**Table 6:** Summary of the features of the chin of FSS or SHS cases from literatures.

Features of the chin	Cases and Figures	Reference	Memo
A crease extending laterally and downward from the corners of the mouth (abbreviated as "CFCM" below) was present in the reported limited number of adult FSS cases.	Case 5, Fig 5	[25]	
	Case 2, Fig 3	[39]	
	Case II8, Fig 2	[40]	
	Case 2, Fig 8 right	[41]	Group 1
	Case 2, Fig 3	[7]	
	Case 1, Fig 1	[42]	
	Case father in Fig 4 and Case of panel 2 in Fig 5	[5]	
	Cases II2, I2 and III1 in supplementary Fig. S2	[23]	
The "CFCM" was also remarkable in most of the reported infants or children with FSS of classical "H shaped dimpling of the chin"(H chin).	Case Renate K., Fig 1	[43]	
	Case 1, Fig 1	[44]	
	Case of kulz, center left in Fig 1	[45]	
	Patient 2, Fig 2	[46]	
	Case 1, Fig 1	[39]	Group 2
	Cases top left 2 and bottom left 2 in Fig 2	[5]	
	Case a, Fig 1	[6]	
	Case 1, Figs 1–4	[47]	
	Case 1, Figs 1 and 2	[48]	
The features of the chin of FSS cases could be described as either "H chin" or "the CFCM".	Case 2, Fig 3	[39]	
	Case 2, Fig 3	[7]	
	Case 1, Fig 1	[42]	Group 3
	Case 1, Figs 1	[49]	
	Cases top center, Fig 2	[5]	
	Case 1, Fig 2	[50]	
The feature of "CFCM" was absent in most of the reported individuals with DA2B.	Cases 1, 2 and 3 (unpublished data)	[11]	
	Cases III-15 and IV-12 in Fig 2; Cases I-1 and III-4 in Fig 4	[51]	Group 4
	Individual V-5 in Fig 2; Individuals I-2 and II-1 in Fig 4	[52]	
	Index case in Fig 1	[53]	
